# Supplementary material for: Rat Hepatic Stellate Cell Line CFSC-2G: Genetic Markers and Short Tandem Repeat Profile Useful for Cell Line Authentication
Source: Cells. 2022 Sep 16;11(18):2900. doi: 10.3390/cells11182900 (PMC9497204; doi:10.3390/cells11182900)
Supplement: Supplementary file 1 [file cells-11-02900-s001.zip › Table S1.pdf]

Table S1. Publications using or mentioning CFSC cells or clones derived thereof

No.	Year	Reference	Clone	Origin of publication
1	1991	Greenwel P, Schwartz M, Rosas M, Peyrol S, Grimaud JA, Rojkind M. Characterization of fat-storing cell lines derived from normal and CCl4-cirrhotic livers. Differences in the production of interleukin-6. Lab Invest. 1991;65(6):644-53. PMID: 1753710	CFSC	USA
2	1993	Ogata I, Sáez CG, Greenwel P, Ponce Mde L, Geerts A, Leinwand LA, Rojkind M. Rat liver fat-storing cell lines express sarcomeric myosin heavy chain mRNA and protein. Cell Motil Cytoskeleton 1993;26(2):125-32. doi: 10.1002/cm.970260204	CFSC-2G, CFSC-3H	USA
3	1993	Greenwel P, Rubin J, Schwartz M, Hertzberg EL, Rojkind M. Liver fat-storing cell clones obtained from a CCl4-cirrhotic rat are heterogeneous with regard to proliferation, expression of extracellular matrix components, interleukin-6, and connexin 43. Lab Invest. 1993;69(2):210-6. PMID: 8394478	CFSC, CFSC-2G, CFSC-8B, CFSC-3H, CFSC-5H	USA
4	1995	Rojkind M, Novikoff PM, Greenwel P, Rubin J, Rojas-Valencia L, de Carvalho AC, Stockert R, Spray D, Hertzberg EL, Wolkoff AW. Characterization and functional studies on rat liver fat-storing cell line and freshly isolated hepatocyte coculture system. Am J Pathol. 1995;146(6):1508-20. PMID: 7778689	CFSC, CFSC-2G, CFSC-8B, CFSC-3H, CFSC-5H	USA
5	1995	Inagaki Y, Truter S, Greenwel P, Rojkind M, Unoura M, Kobayashi K, Ramirez F. Regulation of the alpha 2(I) collagen gene transcription in fat-storing cells derived from a cirrhotic liver. Hepatology 1995;22(2):573-9. PMID: 7635427	CFSC-2G, CFSC-5H	Japan
6	1997	Ogata I, Auster AS, Matsui A, Greenwel P, Geerts A, D'Amico T, Fujiwara K, Kessler E, Rojkind M. Up-regulation of type I procollagen C-proteinase enhancer protein messenger RNA in rats with CCl4-induced liver fibrosis. Hepatology 1997;26(3):611-7. doi: 10.1002/hep.510260312	CFSC, CFSC-2G, CFSC-8B, CFSC-3H, CFSC-5H	USA
7	1997	Fontana L, Jerez D, Rojas-Valencia L, Solís-Herruzo JA, Greenwel P, Rojkind M. Ethanol induces the expression of alpha 1(I) procollagen mRNA in a co-culture system containing a liver stellate cell-line and freshly isolated hepatocytes. Biochim Biophys Acta 1997;1362(2-3):135-44. doi: 10.1016/s0925-4439(97)00056-2	CFSC-2G	USA
8	1998	Okazaki I, Watanabe T, Hozawa S, Maruyama K. Molecular pathology of liver fibrosis. Prog Hepatol 1998;4:23-33.	CFSC-2G, CFSC-5H	Japan
9	1999	Rodríguez-Fragoso L, Alvarez R, Reyes-Esparza JA, Garcés ME. Acetaldehyde increases the activity and gene expression of urokinase type plasminogen activator in a hepatic stellate cell line. Toxicology 1999;137(1):1-11. doi: 10.1016/s0300-483x(99)00064-5	CFSC-2G	Mexico

10	2000	Vasiliou V, Lee J, Pappa A, Petersen DR. Involvement of p65 in the regulation of NF-kappaB in rat hepatic stellate cells during cirrhosis. Biochem Biophys Res Commun. 2000;273(2):546-50. doi: 10.1006/bbrc.2000.2993	NS (CFSC)	USA
11	2000	Reichard JF, Vasiliou V, Petersen DR. Characterization of 4-hydroxy-2-nonenal metabolism in stellate cell lines derived from normal and cirrhotic rat liver. Biochim Biophys Acta 2000;1487(2-3):222-32. doi: 10.1016/s1388-1981(00)00095-0	NS (CFSC?)	USA
12	2001	Gutiérrez-Ruiz MC, Bucio L, Correa A, Souza V, Hernández E, Gómez-Quiroz LE, Kershenobich D. Metadoxine prevents damage produced by ethanol and acetaldehyde in hepatocyte and hepatic stellate cells in culture. Pharmacol Res. 2001;44(5):431-6. doi: 10.1006/phrs.2001.0883	CFSC-2G	Mexico
13	2001	Quiroz SC, Bucio L, Souza V, Hernández E, González E, Gómez-Quiroz L, Kershenobich D, Vargas-Voracková F, Gutiérrez-Ruiz MC. Effect of endotoxin pretreatment on hepatic stellate cell response to ethanol and acetaldehyde. J Gastroenterol Hepatol. 2001;16(11):1267-73. doi: 10.1046/j.1440-1746.2001.02619.x	CFSC-2G	Mexico
14	2001	Inagaki Y, Nemoto T, Nakao A, Dijke Pt, Kobayashi K, Takehara K, Greenwel P. Interaction between GC box binding factors and Smad proteins modulates cell lineage-specific alpha 2(I) collagen gene transcription. J Biol Chem. 2001;276(19):16573-9. doi: 10.1074/jbc.M010485200	CFSC-2G	Japan/The Netherlands/ USA
15	2001	García-Tevijano ER, Berasain C, Rodríguez JA, Corrales FJ, Arias R, Martín-Duce A, Caballería J, Mato JM, Avila MA. Hyperhomocysteinemia in liver cirrhosis: mechanisms and role in vascular and hepatic fibrosis. Hypertension 2001;38(5):1217-21. doi: 10.1161/hy1101.099499	CFSC-2G	Spain
16	2002	del Carmen EM, Souza V, Bucio L, Hernández E, Damián-Matsumura P, Zaga V, Gutiérrez-Ruiz MC. Cadmium induces alpha(1)collagen (I) and metallothionein II gene and alters the antioxidant system in rat hepatic stellate cells. Toxicology 2002;170(1-2):63-73. doi: 10.1016/s0300-483x(01)00531-5	CFSC-2G	Mexico
17	2002	Freeman TL, Kharbanda KK, Tuma DJ, Mailliard ME. Inhibition of hepatic stellate cell collagen synthesis by N-(methylamino)isobutyric acid. Biochem Pharmacol. 2002;63(4):697-706. doi: 10.1016/s0006-2952(01)00885-1	CFSC-2G	USA
18	2002	Hernández E, Correa A, Bucio L, Souza V, Kershenobich D, Gutiérrez-Ruiz MC. Pentoxifylline diminished acetaldehyde-induced collagen production in hepatic stellate cells by decreasing interleukin-6 expression. Pharmacol Res. 2002;46(5):435-43. doi: 10.1016/s1043661802002025	CFSC-2G	Mexico
19	2002	Zhang XL, Liu L, Jiang HQ. Salvia miltiorrhiza monomer IH764-3 induces hepatic stellate cell apoptosis via caspase-3 activation. World J Gastroenterol. 2002;8(3):515-9. doi: 10.3748/wjg.v8.i3.515	CFSC	China
20	2003	Vasiliou V, Qamar L, Pappa A, Sophos NA, Petersen DR. Involvement of the electrophile responsive element and p53 in the activation of hepatic stellate cells as a response to electrophile menadione. Arch Biochem Biophys. 2003;413(2):164-71. doi: 10.1016/s0003-9861(03)00095-x	NS (CFSC)	USA
21	2003	Arnaud A, Fontana L, Angulo AJ, Gil A, López-Pedrosa JM. Proliferation, functionality, and extracellular	CFSC-2G	Spain

		matrix production of hepatocytes and a liver stellate cell line: a comparison between single cultures and cocultures. Dig Dis Sci. 2003;48(7):1406-13. doi: 10.1023/a:1024192100775		
22	2003	Arnaud A, Fontana L, Angulo AJ, Gil A, López-Pedrosa JM. Exogenous nucleosides alter the intracellular nucleotide pool in hepatic cell cultures. Implications in cell proliferation and function. Clin Nutr. 2003;22(4):391-9. doi: 10.1016/s0261-5614(03)00037-2	CFSC-2G	Spain
23	2003	Westhoff JH, Sawitza I, Keski-Oja J, Gressner AM, Breitkopf K. PDGF-BB induces expression of LTBP-1 but not TGF-beta1 in a rat cirrhotic fat storing cell line. Growth Factors 2003;21(3-4):121-30. doi: 10.1080/08977190310001637224	NS (CFSC)	Germany
24	2003	Schaefer B, Rivas-Estilla AM, Meraz-Cruz N, Reyes-Romero MA, Hernández-Nazara ZH, Domínguez-Rosales JA, Schuppan D, Greenwel P, Rojkind M. Reciprocal modulation of matrix metalloproteinase-13 and type I collagen genes in rat hepatic stellate cells. Am J Pathol. 2003;162(6):1771-80. doi: 10.1016/S0002-9440(10)64312-X	CFSC, CFSC-2G, CFSC-3H, CFSC-5H, CFSC-8B	Germany/USA
25	2004	Inagaki Y, Nemoto T, Nakao A. Transcriptional activation of type I collagen gene during hepatic fibrogenesis. In: Extracellular matrix and the liver: Approach to Gene therapy. (Eds. Isao Okazaki, Yosifumi Ninomiya, Scott L. Friedman, and Kyuichi Tanikawa). Academic Press 2003, pp. 233-48. doi: 10.1016/B978-012525251-5/50014-2	CFSC-2G, CFSC-5H	Japan
26	2004	Arnaud A, Fontana L, Sáez-Lara MJ, Gil A, López-Pedrosa JM. Exogenous nucleosides modulate the expression of rat liver extracellular matrix genes in single cultures of primary hepatocytes and a liver stellate cell line and in their co-culture. Clin Nutr. 2004;23(1):43-51. doi: 10.1016/s0261-5614(03)00087-6	CFSC-2G	USA/Spain
27*	2004	Xu XB, Leng XS, He ZP, Liang ZQ, Lin K, Yu X, Wei YH. [Effects of anti-sense Smad4 gene on the biological characteristics of the fat-storing cell line CFSC]. Zhonghua Yi Xue Za Zhi. 2004;84(7):587-91. PMID: 15144596	NS (CFSC?)	China
28	2004	Souza V, Escobar Mdel C, Bucio L, Hernández E, Gutiérrez-Ruiz MC. Zinc pretreatment prevents hepatic stellate cells from cadmium-produced oxidative damage. Cell Biol Toxicol. 2004;20(4):241-51. doi: 10.1023/b:cbto.0000038462.39859.2f	CFSC-2G	Mexico
29	2004	Freeman TL, Thiele GM, Klassen LW, Klassen BT, Mailliard ME. N-(methylamino)isobutyric acid inhibits proliferation of CFSC-2C hepatic stellate cells. Biochem Pharmacol. 2004;68(2):223-30. doi: 10.1016/j.bcp.2004.03.012	CFSC-2G**	USA
30	2004	Bolkenius U, Hahn D, Gressner AM, Breitkopf K, Dooley S, Wickert L. Glucocorticoids decrease the bioavailability of TGF-beta which leads to a reduced TGF-beta signaling in hepatic stellate cells. Biochem Biophys Res Commun. 2004;325(4):1264-70. doi: 10.1016/j.bbrc.2004.10.164	NS (CFSC?)	Germany
31	2004	Wang YF, Nan X, Zhang R, Li YH, Yue W, Yan F, Pei XT. Differentiation of bone marrow derived Thy-1(+)beta M-2(-) cells into hepatocytes induced by coculture with transgenic CFSCs. Chin Sci Bull 2004;49:889-94. doi: 10.1007/BF03184005	NS (CFSC?)	China

32*	2005	Wang YF, Nan X, Li YH, Zhang R, Yue W, Yan F, Pei XT. [Sustaining effect of gene-transferring hepatic stellate cell strain CFSC/HGF on hepatocytes development]. Zhonghua Gan Zang Bing Za Zhi. 2005;13(1):45-8. PMID: 15670492	NS (CSFC?)	China
33	2005	Wang Y, Nan X, Li Y, Zhang R, Yue W, Yan F, Pei X. Induction of umbilical cord blood-derived beta2m-c-Met+ cells into hepatocyte-like cells by coculture with CFSC/HGF cells. Liver Transpl. 2005;11(6):635-43. doi: 10.1002/lt.20419	NS (CFSC?)	China
34*	2005	Wang YF, Xue YL, Nan X, Liang F, Luo Y, Li YL, Gao YH, Yue W, Pei XT. [Sustainment of hepatocyte function with mixed cellular co-encapsulation]. Zhonghua Yi Xue Za Zhi. 2005;85(35):2481-6. PMID: 16321274	NS (CFSC?)	China
35	2005	Schulze-Krebs A, Preimel D, Popov Y, Bartenschlager R, Lohmann V, Pinzani M, Schuppan D. Hepatitis C virus-replicating hepatocytes induce fibrogenic activation of hepatic stellate cells. Gastroenterology 2005;129(1):246-58. doi: 10.1053/j.gastro.2005.03.089	CFSC-2G	Germany/USA/Italy
36	2006	Dooley S, Said HM, Gressner AM, Floege J, En-Nia A, Mertens PR. Y-box protein-1 is the crucial mediator of antifibrotic interferon-gamma effects. J Biol Chem. 2006;281(3):1784-95. doi: 10.1074/jbc.M510215200	CFSC-2G	Germany
37	2007	de Villiers WJ, Song Z, Nasser MS, Deaciuc IV, McClain CJ. 4-Hydroxynonenal-induced apoptosis in rat hepatic stellate cells: mechanistic approach. J Gastroenterol Hepatol. 2007;22(3):414-22. doi: 10.1111/j.1440-1746.2006.04625.x	CFSC-2G	USA
38	2007	Sun Y, Fan J, Shen H, Li P, Cattini P, Gong Y. Cloning and promoter activity of rat Smad1 5'-flanking region in rat hepatic stellate cells. Mol Cell Biochem. 2007;304(1-2):227-34. doi: 10.1007/s11010-007-9504-8	CFSC-8B	Canada/China
39	2007	Shen H, Fan J, Burczynski F, Minuk GY, Cattini P, Gong Y. Increased Smad1 expression and transcriptional activity enhances trans-differentiation of hepatic stellate cells. J Cell Physiol. 2007;212(3):764-70. doi: 10.1002/jcp.21074	CFSC-8B	Canada/China
40*	2007	Shi LJ, Li SX, Sun B, Wang JH, Li HL, Jin LH. [Effects of bone marrow mesenchymal stem cells on the proliferation of hepatocytes and cirrhotic fat-storing cells in vitro]. Zhonghua Gan Zang Bing Za Zhi. 2007;15(9):681-4. PMID: 17903371	NS (CFSC)	China
41	2007	Herrmann J, Gressner AM, Weiskirchen R. Immortal hepatic stellate cell lines: useful tools to study hepatic stellate cell biology and function? J Cell Mol Med. 2007;11(4):704-22. doi: 10.1111/j.1582-4934.2007.00060.x	CFSC-2G	Germany
42	2008	Hernández E, Bucio L, Souza V, Escobar MC, Gómez-Quiroz LE, Farfán B, Kershenobich D, Gutiérrez-Ruiz MC. Pentoxifylline downregulates alpha (I) collagen expression by the inhibition of Ikappabalpha degradation in liver stellate cells. Cell Biol Toxicol. 2008;24(4):303-14. doi: 10.1007/s10565-007-9039-5	CFSC-2G	Mexico
43	2008	Inagaki Y, Higashi K, Kushida M, Hong YY, Nakao S, Higashiyama R, Moro T, Itoh J, Mikami T, Kimura T, Shiota G, Kuwabara I, Okazaki I. Hepatocyte growth factor suppresses profibrogenic signal	CFSC-2G	Japan/USA

		transduction via nuclear export of Smad3 with galectin-7. Gastroenterology 2008;134(4):1180-90. doi: 10.1053/j.gastro.2008.01.014		
44	2008	Ohayon O, Mawasi N, Pevzner A, Tryvitz A, Gildor T, Pines M, Rojkind M, Paizi M, Spira G. Halofuginone upregulates the expression of heparanase in thioacetamide-induced liver fibrosis in rats. Lab Invest. 2008;88(6):627-33. doi: 10.1038/labinvest.2008.30	CFSC-3H, CFSC-8B	Israel/USA
45	2008	Camino AM, Atorrasagasti C, Maccio D, Prada F, Salvatierra E, Rizzo M, Alaniz L, Aquino JB, Podhajcer OL, Silva M, Mazzolini G. Adenovirus-mediated inhibition of SPARC attenuates liver fibrosis in rats. J Gene Med. 2008;10(9):993-1004. doi: 10.1002/jgm.1228	CFSC-2G	Argentina
46	2008	Maubach G, Lim MC, Zhuo L. Nuclear cathepsin F regulates activation markers in rat hepatic stellate cells. Mol Biol Cell. 2008;19(10):4238-48. doi: 10.1091/mbc.e08-03-0291	CFSC-8B	Singapore
47	2009	Sun X, Zhang XD, Cheng G, Hu YH, Wang HY. Inhibition of hepatic stellate cell proliferation by heat shock protein 90 inhibitors in vitro. Mol Cell Biochem. 2009;330(1-2):181-5. doi: 10.1007/s11010-009-0131-4	NS (CFSC clone?)	China
48	2009	Sun X, Zhang X, Hu H, Lu Y, Chen J, Yasuda K, Wang H. Berberine inhibits hepatic stellate cell proliferation and prevents experimental liver fibrosis. Biol Pharm Bull. 2009;32(9):1533-7. doi: 10.1248/bpb.32.1533	NS (CFSC?)	China
49	2009	Szuster-Ciesielska A, Plewka K, Daniluk J, Kandefer-Szerszeń M. Zinc supplementation attenuates ethanol- and acetaldehyde-induced liver stellate cell activation by inhibiting reactive oxygen species (ROS) production and by influencing intracellular signaling. Biochem Pharmacol. 2009;78(3):301-14. doi: 10.1016/j.bcp.2009.04.009	CFSC-2G	Poland
50	2009	Ruehl M, Erben U, Kim K, Freise C, Dagdelen T, Eisele S, Trowitzsch-Kienast W, Zeitz M, Jia J, Stickel F, Somasundaram R. Extracts of Lindera obtusiloba induce antifibrotic effects in hepatic stellate cells via suppression of a TGF-beta-mediated profibrotic gene expression pattern. J Nutr Biochem. 2009;20(8):597-606. doi: 10.1016/j.jnutbio.2008.06.003	CFSC-2G	Germany
51	2009	Enami Y, Bandi S, Kapoor S, Krohn N, Joseph B, Gupta S. Hepatic stellate cells promote hepatocyte engraftment in rat liver after prostaglandin-endoperoxide synthase inhibition. Gastroenterology 2009;136(7):2356-64. doi: 10.1053/j.gastro.2009.03.003	CFSC-8B	Japan/USA
52	2010	González-Puertos VY, Hernández-Pérez E, Nuño-Lámbarri N, Ventura-Gallegos JL, López-Diázguerrero NE, Robles-Díaz G, Gutiérrez-Ruiz MC, Konigsberg M. Bcl-2 overexpression in hepatic stellate cell line CFSC-2G, induces a pro-fibrotic state. J Gastroenterol Hepatol. 2010;25(7):1306-14. doi: 10.1111/j.1440-1746.2009.06175.x	CFSC-2G	Mexico
53	2010	Ye Y, Dan Z. All-trans retinoic acid diminishes collagen production in a hepatic stellate cell line via suppression of active protein-1 and c-Jun N-terminal kinase signal. J Huazhong Univ Sci Technolog Med Sci. 2010;30(6):726-33. doi: 10.1007/s11596-010-0648-5	CFSC-2G	China

54	2011	Atorrasagasti C, Aquino JB, Hofman L, Alaniz L, Malvicini M, Garcia M, Benedetti L, Friedman SL, Podhajcer O, Mazzolini G. SPARC downregulation attenuates the profibrogenic response of hepatic stellate cells induced by TGF-β1 and PDGF. Am J Physiol Gastrointest Liver Physiol. 2011;300(5):G739-48. doi: 10.1152/ajpgi.00316.2010	CFSC-2G	Argentina/USA
55	2011	Kastanis GJ, Hernandez-Nazara Z, Nieto N, Rincón-Sanchez AR, Popratiloff A, Dominguez-Rosales JA, Lechuga CG, Rojkind M. The role of dystroglycan in PDGF-BB-dependent migration of activated hepatic stellate cells/myofibroblasts. Am J Physiol Gastrointest Liver Physiol. 2011;301(3):G464-74. doi: 10.1152/ajpgi.00078.2011	CFSC-2G, CFSC-8B	USA/Mexico/Spa in
56	2011	Mòdol T, Natal C, Pérez de Obanos MP, Domingo de Miguel E, Iraburu MJ, López-Zabalza MJ. Apoptosis of hepatic stellate cells mediated by specific protein nitration. Biochem Pharmacol. 2011;81(3):451-8. doi: 10.1016/j.bcp.2010.10.017	CFSC-2G	Spain
57	2012	Borkham-Kamphorst E, van Roeyen CR, Van de Leur E, Floege J, Weiskirchen R. CCN3/NOV small interfering RNA enhances fibrogenic gene expression in primary hepatic stellate cells and cirrhotic fat storing cell line CFSC. J Cell Commun Signal. 2012;6(1):11-25. doi: 10.1007/s12079-011-0141-3	CFSC(-2G)	Germany
58	2012	Fan J, Shen H, Dai Q, Burzynski FJ, Minuk GY, Gong Y. Extent of extracellular signal-regulated kinases phosphorylation determines the sensitivity of hepatic stellate cells to staurosporine-induced apoptosis. Zhong Nan Da Xue Xue Bao Yi Xue Ban. 2012;37(1):11-6. doi: 10.3969/j.issn.1672-7347.2012.01.003	CFSC-8B, CFSC-2G, CFSC-3H, CFSC-5H	Canada/China
59	2012	Lim JY, Oh MA, Kim WH, Sohn HY, Park SI. AMP-activated protein kinase inhibits TGF-β-induced fibrogenic responses of hepatic stellate cells by targeting transcriptional coactivator p300. J Cell Physiol. 2012;227(3):1081-9. doi: 10.1002/jcp.22824	CFSC-2G	Korea
60	2012	Piccioni F, Malvicini M, Garcia MG, Rodriguez A, Atorrasagasti C, Kippes N, Piedra Buena IT, Rizzo MM, Bayo J, Aquino J, Viola M, Passi A, Alaniz L, Mazzolini G. Antitumor effects of hyaluronic acid inhibitor 4-methylumbelliferone in an orthotopic hepatocellular carcinoma model in mice. Glycobiology 2012;22(3):400-10. doi: 10.1093/glycob/cwr158	CFSC-2G	Argentina/Italy
61	2012	Boaru SG, Borkham-Kamphorst E, Tihaa L, Haas U, Weiskirchen R. Expression analysis of inflammasomes in experimental models of inflammatory and fibrotic liver disease. J Inflamm (Lond). 2012;9(1):49. doi: 10.1186/1476-9255-9-49	CFSC-2G	Germany
62	2012	Madsen DH, Jürgensen HJ, Ingvarsen S, Melander MC, Vainer B, Egerod KL, Hald A, Rønø B, Madsen CA, Bugge TH, Engelholm LH, Behrendt N. Endocytic collagen degradation: a novel mechanism involved in protection against liver fibrosis. J Pathol. 2012;227(1):94-105. doi: 10.1002/path.3981	CFSC-2G	Denmark/USA
63	2012	Zong L, Qu Y, Xu My, Dong Yw, Lu Lg. 18α-Glycyrrhetinic acid down-regulates expression of type I and III collagen via TGF-B1/Smad signaling pathway in human and rat hepatic stellate cells. Int J Med Sci 2012; 9(5):370-9. doi:10.7150/ijms.4395	CFSC	China

64	2013	Peng Y, Yang H, Zhu T, Zhao M, Deng Y, Liu B, Shen H, Hu G, Wang Z, Tao L. The antihepatic fibrotic effects of fluorofenidone via MAPK signalling pathways. Eur J Clin Invest. 2013;43(4):358-68. doi: 10.1111/eci.12053	CFSC-2G	China
65	2013	von Schönfels W, von Kampen O, Patsenker E, Stickel F, Schniewind B, Hinz S, Ahrens M, Balschun K, Egberts JH, Richter K, Landrock A, Sipos B, Will O, Huebbe P, Schreiber S, Nothnagel M, Röcken C, Rimbach G, Becker T, Hampe J, Schafmayer C. Metabolic signature of electrosurgical liver dissection. PLoS One 2013;8(9):e72022. doi: 10.1371/journal.pone.0072022	CFSC-2G	Germany/Switzerland
66	2013	Meurer SK, Alsamman M, Sahin H, Wasmuth HE, Kisseleva T, Brenner DA, Trautwein C, Weiskirchen R, Scholten D. Overexpression of endoglin modulates TGF-β1-signalling pathways in a novel immortalized mouse hepatic stellate cell line. PLoS One 2013;8(2):e56116. doi: 10.1371/journal.pone.0056116	CFSC-2G	Germany/USA
67	2013	Szuster-Ciesielska A, Mizerska-Dudka M, Daniluk J, Kandefer-Szerszeń M. Butein inhibits ethanol-induced activation of liver stellate cells through TGF-β, NFκB, p38, and JNK signaling pathways and inhibition of oxidative stress. J Gastroenterol. 2013;48(2):222-37. doi: 10.1007/s00535-012-0619-7	CFSC-2G	Poland
68	2013	Calleja MA, Vieites JM, Montero-Meléndez T, Torres MI, Faus MJ, Gil A, Suárez A. The antioxidant effect of β-caryophyllene protects rat liver from carbon tetrachloride-induced fibrosis by inhibiting hepatic stellate cell activation. Br J Nutr. 2013;109(3):394-401. doi: 10.1017/S0007114512001298	CFSC-2G	Spain/UK
69	2013	Liu Y, Liu H, Meyer C, Li J, Nadalin S, Königsrainer A, Weng H, Dooley S, Ten Dijke P. Transforming growth factor-β (TGF-β)-mediated connective tissue growth factor (CTGF) expression in hepatic stellate cells requires Stat3 signaling activation. J Biol Chem. 2013;288(42):30708-19. doi: 10.1074/jbc.M113.478685	CFSC-2G	Germany/The Netherlands/China
70	2014	Bahde R, Kapoor S, Gupta S. Nonselective inhibition of prostaglandin-endoperoxide synthases by naproxen ameliorates acute or chronic liver injury in animals. Exp Mol Pathol. 2014;96(1):27-35. doi: 10.1016/j.yexmp.2013.10.017	CFSC-8B	Germany/USA
71	2014	Geng Y, Wang J, Xie M, Lu Z, Xu H, Shi JS, Xu ZH. Screening and isolation for anti-hepatofibrotic components from medicinal mushrooms using TGF-β1-induced live fibrosis in hepatic stellate cells. Int J Med Mushrooms 2014;16(6):529-39. doi: 10.1615/intjmedmushrooms.v16.i6.30	CFSC-8B	China
72	2014	Bahde R, Kapoor S, Viswanathan P, Spiegel HU, Gupta S. Endothelin-1 receptor A blocker darusentan decreases hepatic changes and improves liver repopulation after cell transplantation in rats. Hepatology 2014;59(3):1107-17. doi: 10.1002/hep.26766	CFSC-8B	Germany/USA
73	2014	Duval F, Moreno-Cuevas JE, González-Garza MT, Rodríguez-Montalvo C, Cruz-Vega DE. Liver fibrosis and protection mechanisms action of medicinal plants targeting apoptosis of hepatocytes and hepatic stellate cells. Adv Pharmacol Sci. 2014;2014:373295. doi: 10.1155/2014/373295	CFSC	Mexico
74	2014	Li J, Pan Y, Kan M, Xiao X, Wang Y, Guan F, Zhang X, Chen L. Hepatoprotective effects of berberine on liver fibrosis via activation of AMP-activated protein kinase. Life Sci. 2014;98(1):24-30. doi:	CFSC-2G	China

		10.1016/j.lfs.2013.12.211		
75	2015	Wang X, Zhao W, Wang J, Shi K, Qin X, Kong Q, Wang G, Mu L, Li H, Sun B, Shi L. Bone marrow stromal cells inhibit the activation of liver cirrhotic fat-storing cells via adrenomedullin secretion. Dig Dis Sci. 2015;60(5):1325-34. doi: 10.1007/s10620-014-3423-9	NS (CFSC?)	China
76	2015	Chen L, Li L, Chen J, Li L, Zheng Z, Ren J, Qiu Y. Oleoylethanolamide, an endogenous PPAR-α ligand, attenuates liver fibrosis targeting hepatic stellate cells. Oncotarget 2015;6(40):42530-40. doi: 10.18632/oncotarget.6466	CFSC	China/USA
77	2016	Geng Y, Wang J, Sun Q, Xie M, Lu ZM, Xu HY, Shi JS, Xu ZH. Identification of Antrodin B from *Antrodia camphorata* as a new anti-hepatofibrotic compound using a rapid cell screening method and biological evaluation. Hepatol Res. 2016;46(3):E15-25. doi: 10.1111/hepr.12516	CFSC-8B	China
78	2016	Borkham-Kamphorst E, Steffen BT, Van de Leur E, Haas U, Tihaa L, Friedman SL, Weiskirchen R. CCN1/CYR61 overexpression in hepatic stellate cells induces ER stress-related apoptosis. Cell Signal 2016;28(1):34-42. doi: 10.1016/j.cellsig.2015.10.013	CFSC-2G	Germany/USA
79	2016	Fiore E, Malvicini M, Bayo J, Peixoto E, Atorrasagasti C, Sierra R, Rodríguez M, Gómez Bustillo S, García MG, Aquino JB, Mazzolini G. Involvement of hepatic macrophages in the antifibrotic effect of IGF-I-overexpressing mesenchymal stromal cells. Stem Cell Res Ther. 2016;7(1):172. doi: 10.1186/s13287-016-0424-y	CFSC-2G	Argentina
80	2016	Wang X, Wu X, Zhang A, Wang S, Hu C, Chen W, Shen Y, Tan R, Sun Y, Xu Q. Targeting the PDGF-B/PDGFR-β interface with Destruxin A5 to selectively block PDGF-BB/PDGFR-ββ signaling and attenuate liver fibrosis. EBioMedicine 2016;7:146-56. doi: 10.1016/j.ebiom.2016.03.042	CFSC-8B	China
81	2017	Geng Y, Sun Q, Li W, Lu ZM, Xu HY, Shi JS, Xu ZH. The common dietary flavonoid myricetin attenuates liver fibrosis in carbon tetrachloride treated mice. Mol Nutr Food Res. 2017;61(4). doi: 10.1002/mnfr.201600392	CFSC-8B	China
82	2017	Labine M, Gong Y, Minuk GY. Long-term, low-dose exposure to microcystin-LR does not cause or increase the severity of liver disease in rodents. Ann Hepatol 2017; 16: 959-65. doi:10.5604/01.3001.0010.5288	CFSC-2G	Canada
83	2017	Qiu SS, He Y, Li SL, Peng Y, Yang CY, Peng XQ, Gan QX, Zheng LF, Zhang J, Yang HX, Tao LJ. Peroxiredoxin-1 attenuates hepatic fibrosis by inhibiting TGF-beta 1/Smad3 and ROS signal pathways. Int J Clin Exp Med 2017;10:8896-8906. ISSN:1940-5901/IJCEM0050241	CFSC-2G	China
84	2019	Chen H, Gan Q, Yang C, Peng X, Qin J, Qiu S, Jiang Y, Tu S, He Y, Li S, Yang H, Tao L, Peng Y. A novel role of glutathione S-transferase A3 in inhibiting hepatic stellate cell activation and rat hepatic fibrosis. J Transl Med. 2019;17(1):280. doi: 10.1186/s12967-019-2027-8 Erratum in: J Transl Med. 2020;18(1):182.	CFSC-2G	China
85	2019	Zhang AH, Jiang N, Wang XQ, Tan RX. Galewone, an anti-fibrotic polyketide from *Daldinia eschscholzii* with an undescribed carbon skeleton. Sci Rep. 2019;9(1):14316. doi: 10.1038/s41598-019-50868-9	CFSC-8B	China

86	2020	Wang X, Gao Y, Li Y, Huang Y, Zhu Y, Lv W, Wang R, Gou L, Cheng C, Feng Z, Xie J, Tian J, Yao R. Roseotoxin B alleviates cholestatic liver fibrosis through inhibiting PDGF-B/PDGFR-β pathway in hepatic stellate cells. Cell Death Dis. 2020;11(6):458. doi: 10.1038/s41419-020-2575-0	CFSC-8B	China
87	2020	Wang X. Study on the effects and mechanism of matrine on proliferation and collagen synthesis of hepatic stellate cells CFSC-8B activated by acetaldehyde. China Pharmacy 2020;12:1353-8.	CFSC-8B	China
88	2021	Freise C, Lee H, Chronowski C, Chan D, Cziomer J, Rühl M, Dagdelen T, Lösekann M, Erben U, Catic A, Tegge W, Schuppan D, Somasundaram R, Sahin E. Alpha-single chains of collagen type VI inhibit the fibrogenic effects of triple helical collagen VI in hepatic stellate cells. PLoS One 2021;16(9):e0254557. doi: 10.1371/journal.pone.0254557	CFSC	Germany/USA
89	2022	Weiz G, Molejon MI, Malvicini M, Sukowati CHC, Tiribelli C, Mazzolini G, Breccia JD. Glycosylated 4-methylumbelliferone as a targeted therapy for hepatocellular carcinoma. Liver Int. 2022;42(2):444-57. doi: 10.1111/liv.15084	CFSC-2G	Argentina/Italy
90	2022	Xu XY, Geng Y, Xu HX, Ren Y, Liu DY, Mao Y. *Antrodia camphorata*-derived Antrodin C inhibits liver fibrosis by blocking TGF-Beta and PDGF signaling pathways. Front Mol Biosci. 2022;9:835508. doi: 10.3389/fmolb.2022.835508	CFSC-8B	China
91	2022	Tanaka T, Moriya K, Tsunenaga M, Yanagawa T, Morita H, Minowa T, Tagawa YI, Hanagata N, Inagaki Y, Ikoma T. Visualized procollagen Iα1 demonstrates the intracellular processing of propeptides. Life Sci Alliance 2022;5(5):e202101060. doi: 10.26508/lsa.202101060	CFSC-2G, CFSC-5H	Japan
92	2022	Schröder SK, Schüler HM, Petersen KV, Tesauro C, Knudsen BR, Pedersen FS, Krus F, Buhl EM, Roeb E, Roderfeld M, Borojevic R, Almeida JL, Weiskirchen R. Genetic and molecular characterization of the immortalized murine hepatic stellate cell line GRX. Cells 2022;11(9):1504. doi: 10.3390/cells11091504	CFSC-2G	Germany/Denmark/Brazil/USA

* Article written in Chinese; ** Supposed error (i.e., CFSC-2C) in title or publication.
