# Supplementary figures and images for: Rat Hepatic Stellate Cell Line CFSC-2G: Genetic Markers and Short Tandem Repeat Profile Useful for Cell Line Authentication
Source: Cells. 2022 Sep 16;11(18):2900. doi: 10.3390/cells11182900 (PMC9497204; doi:10.3390/cells11182900)

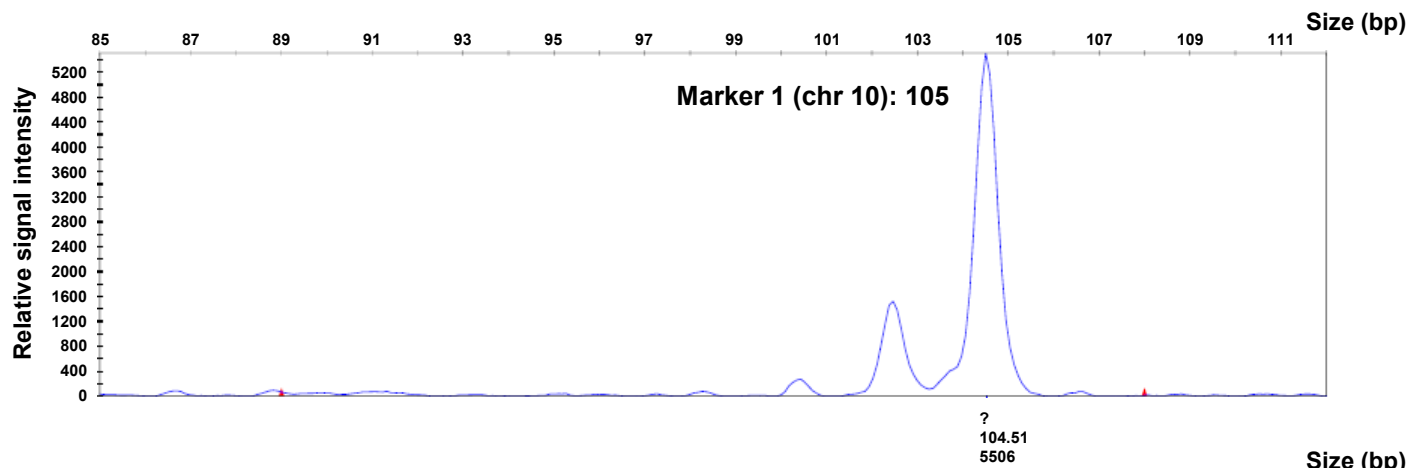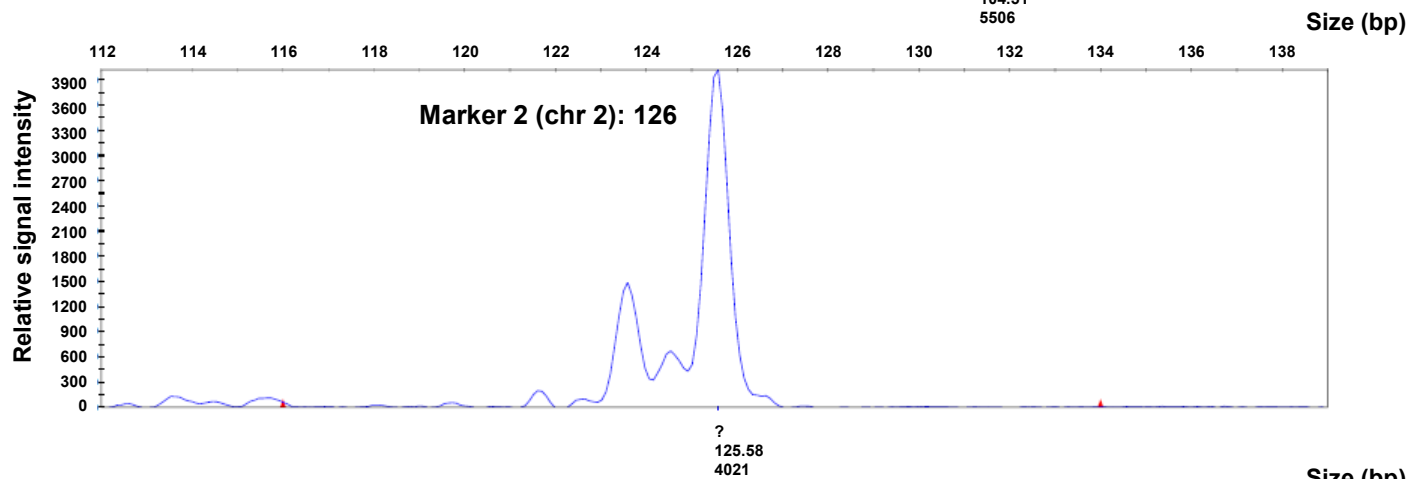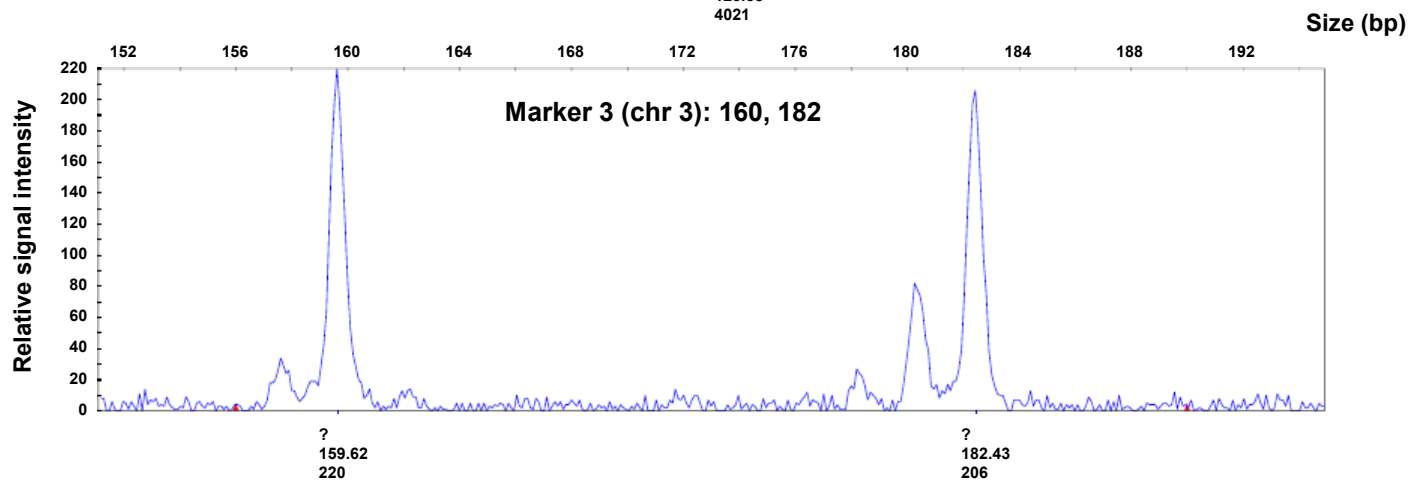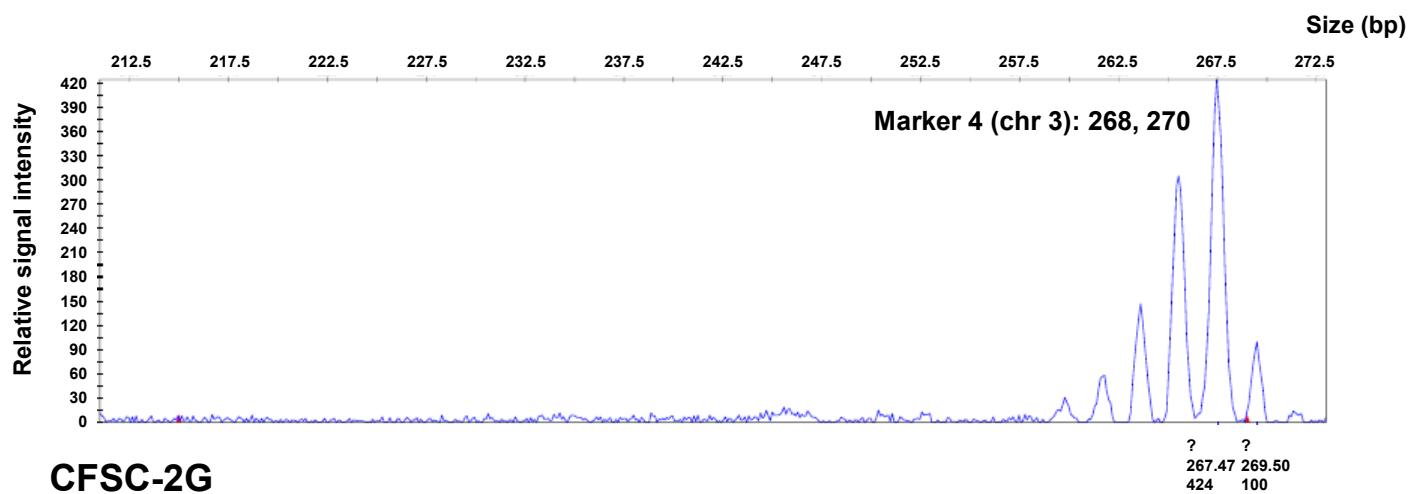

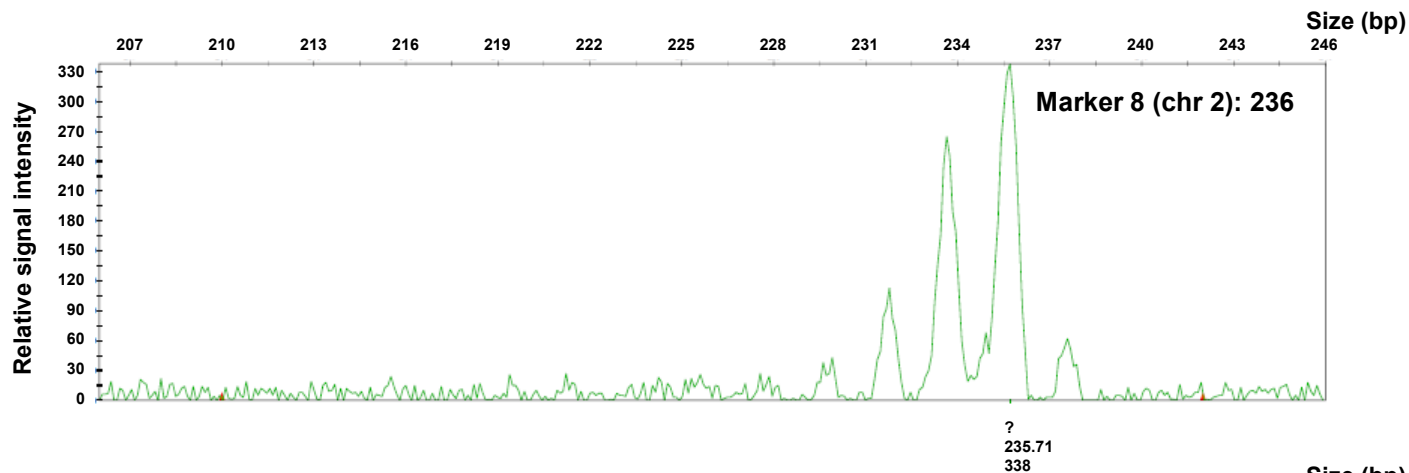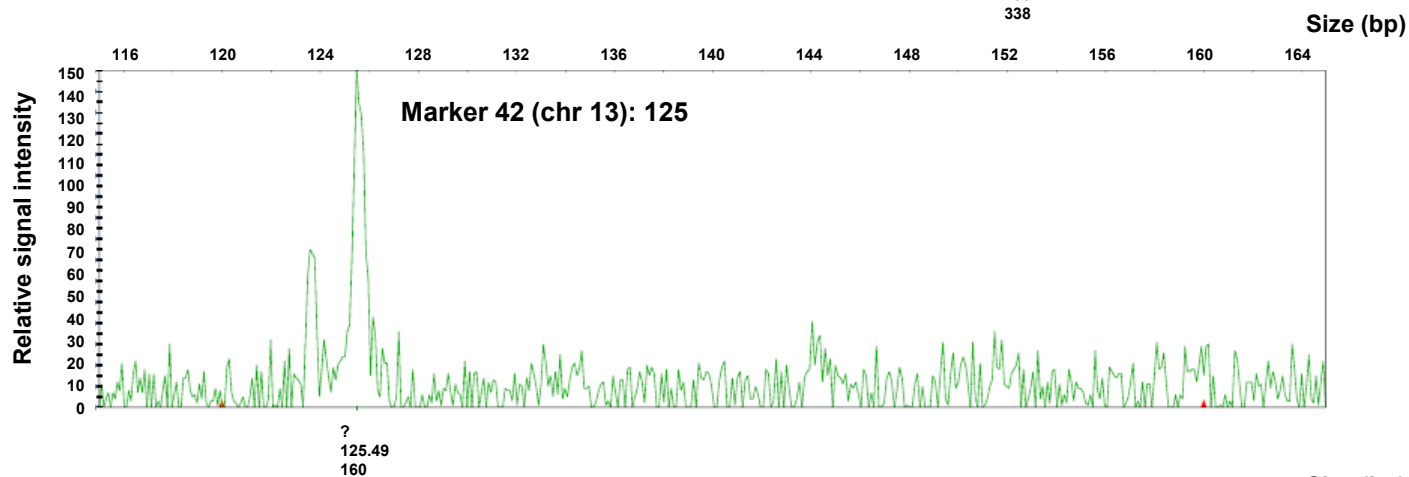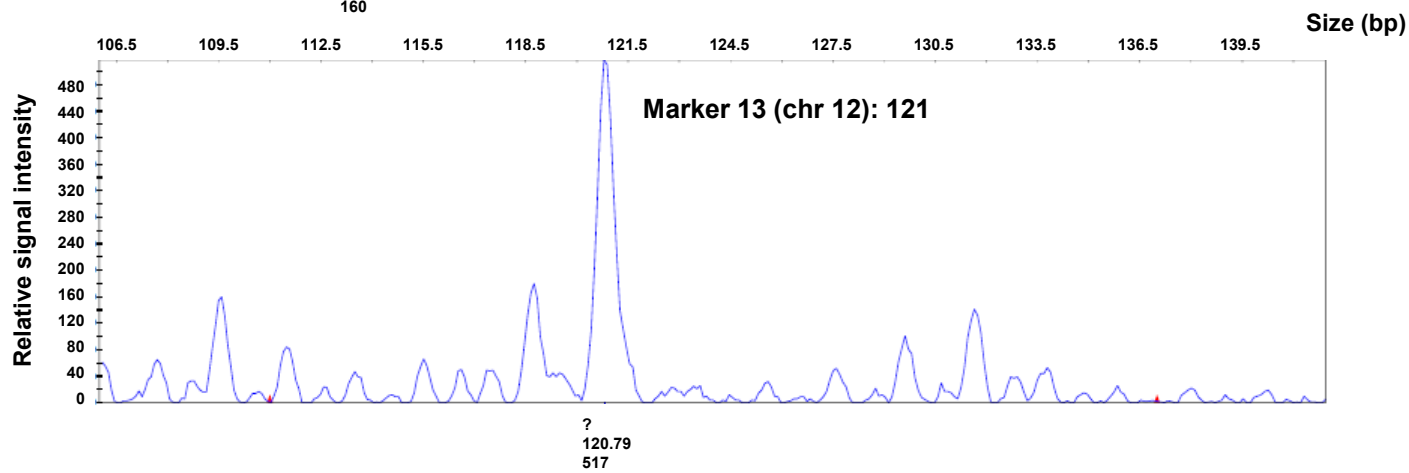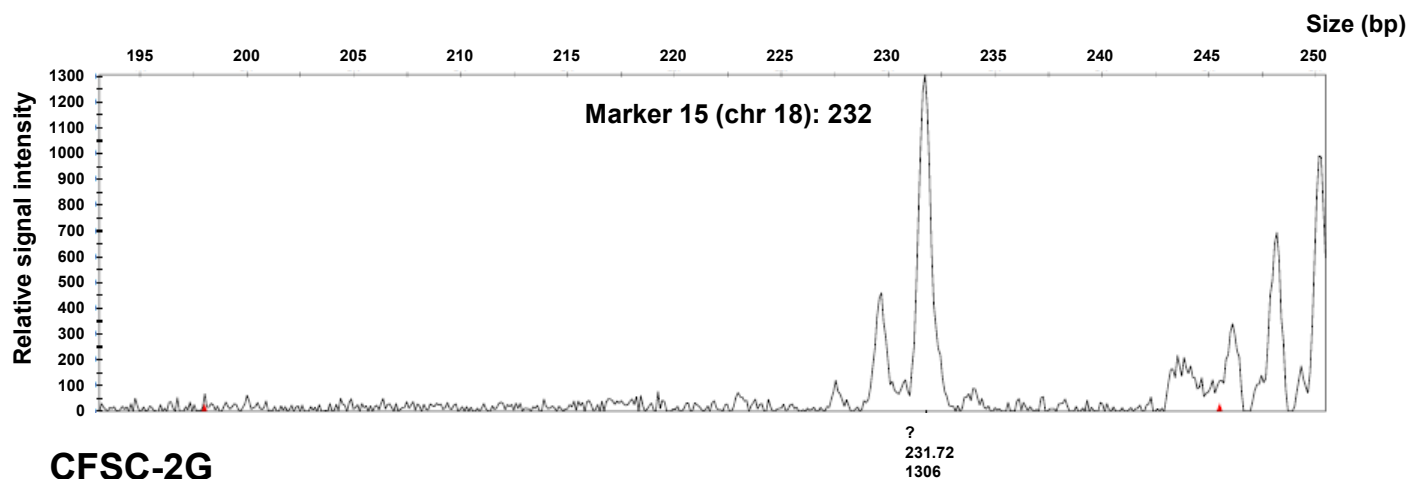

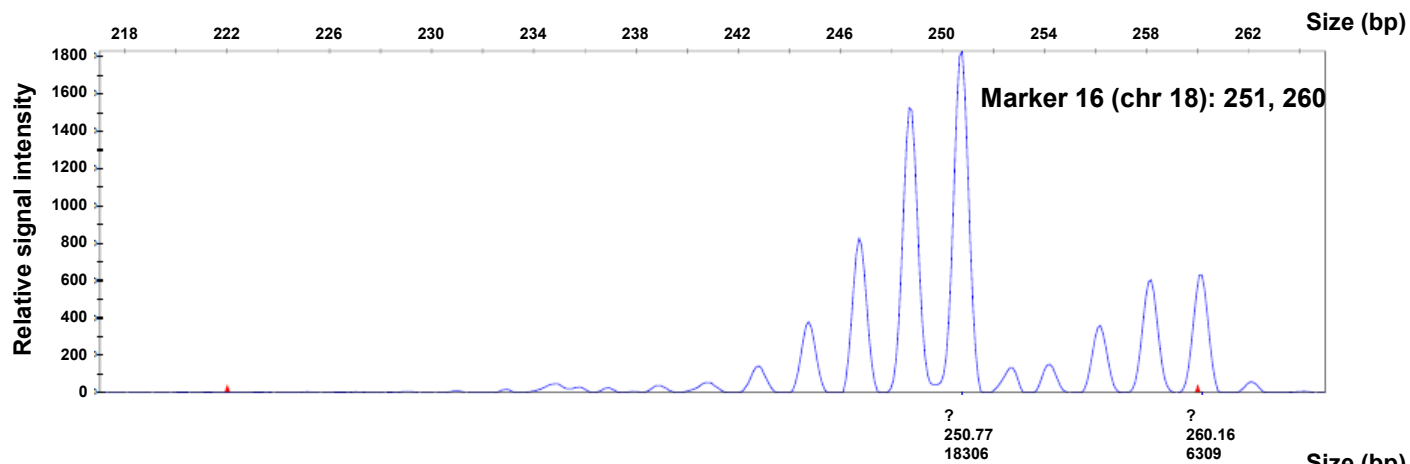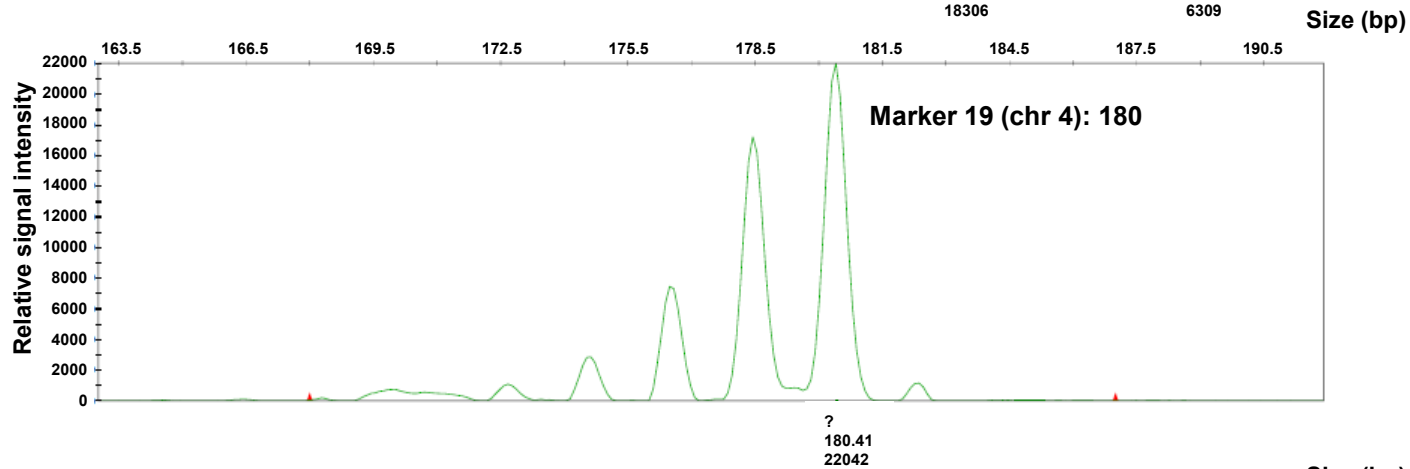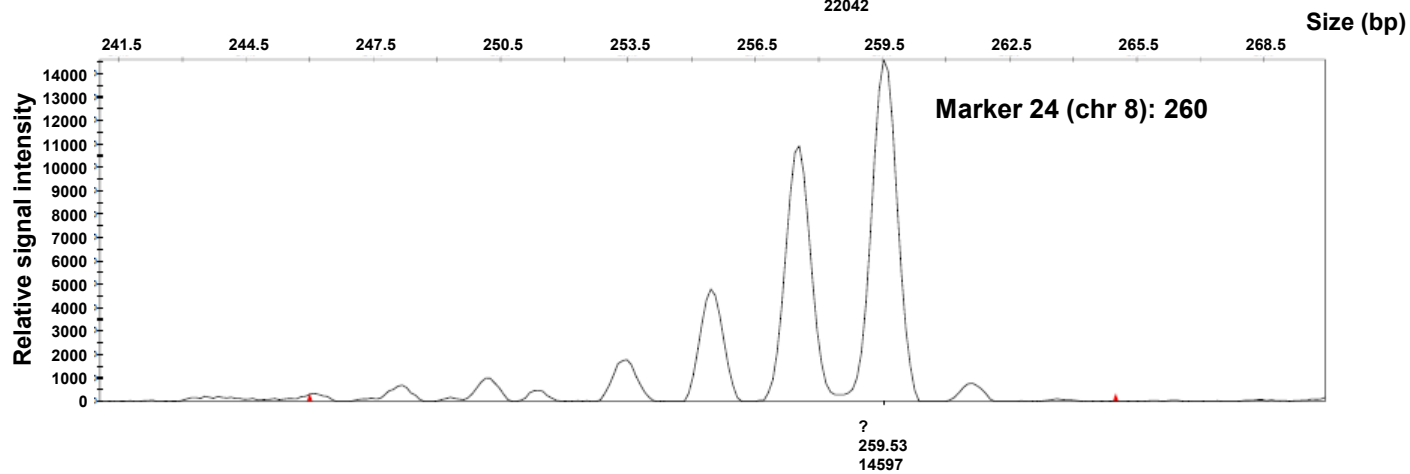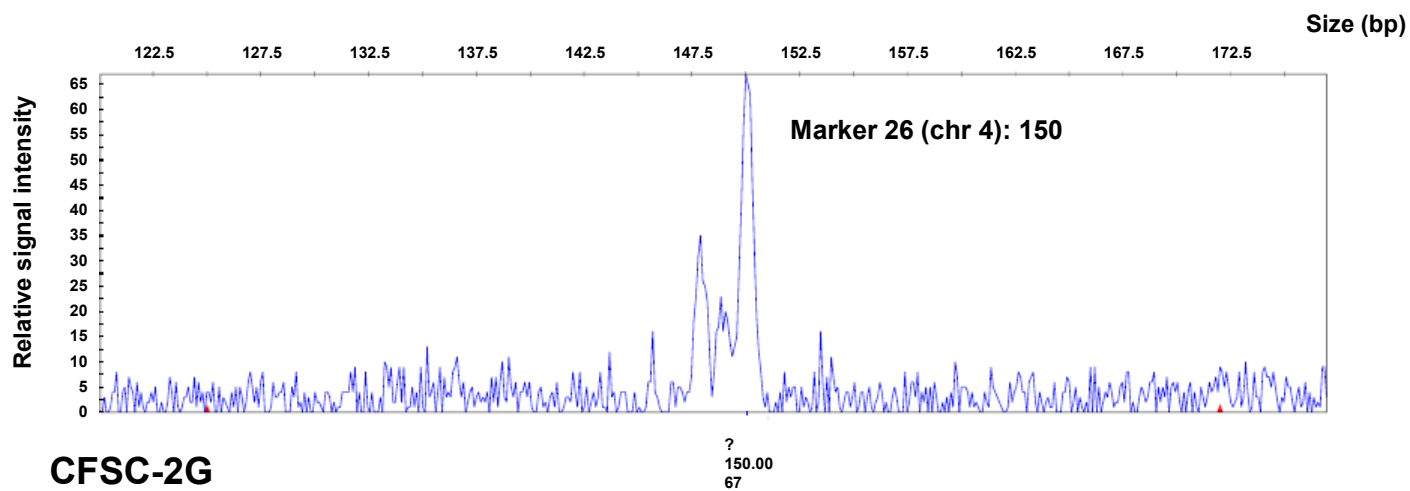

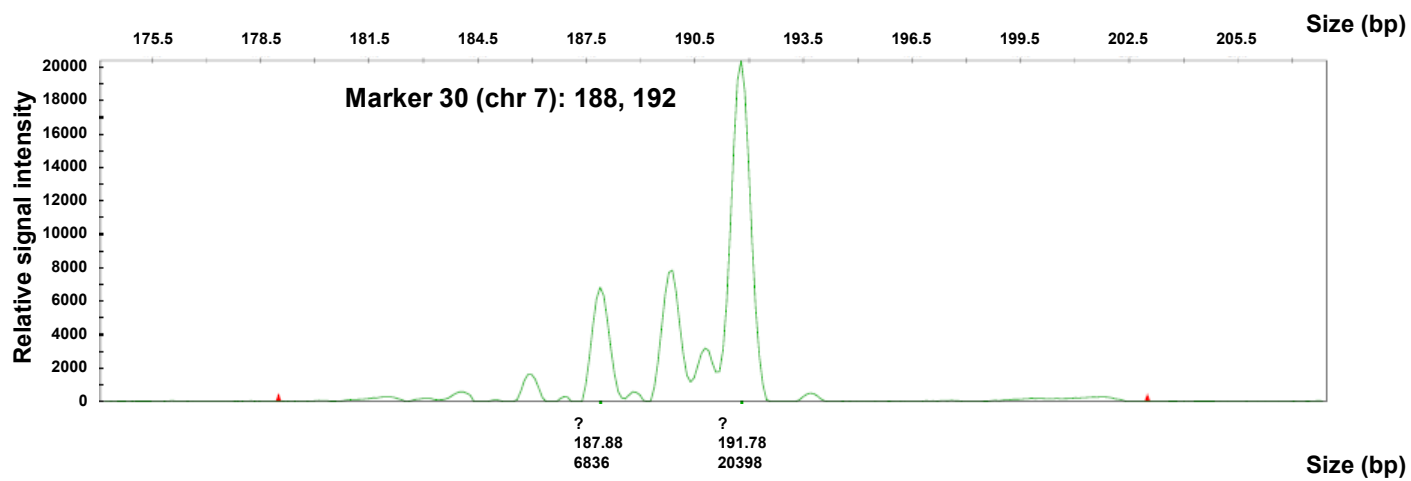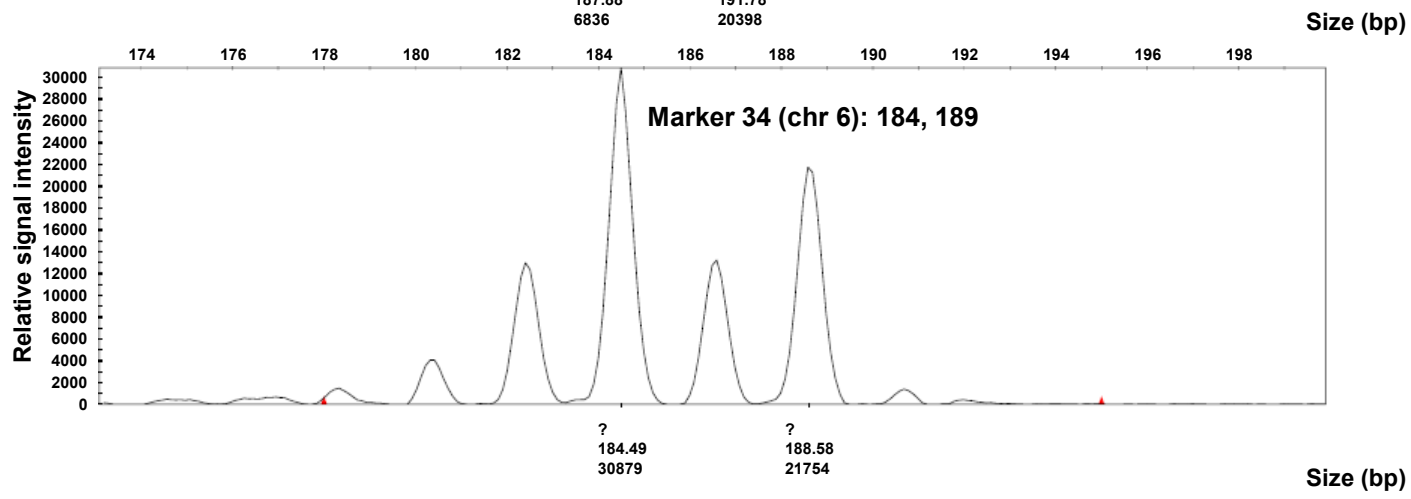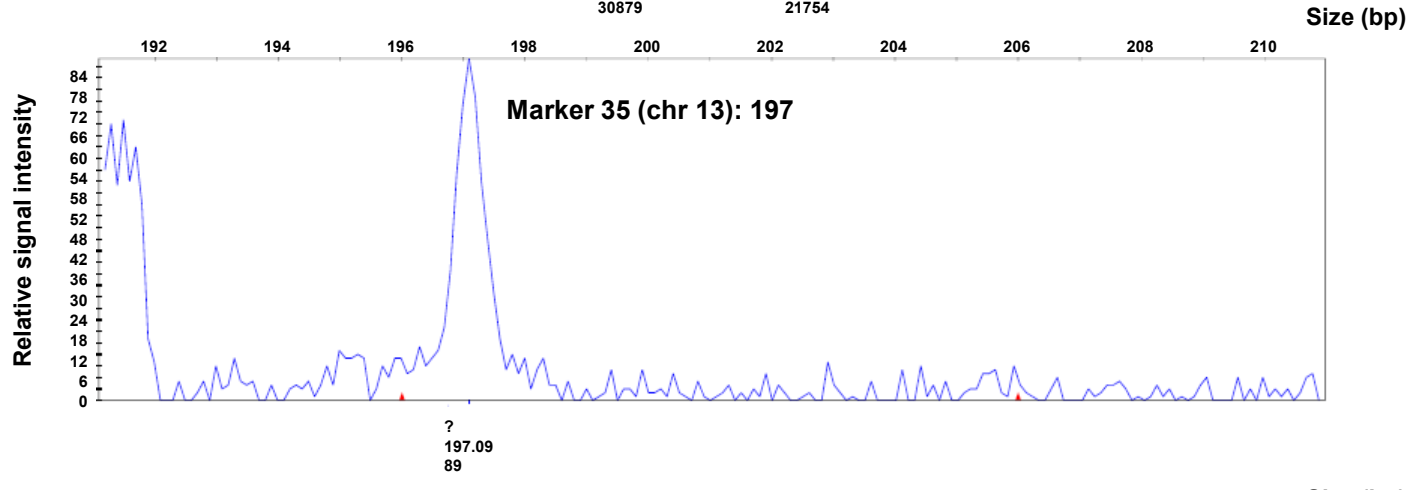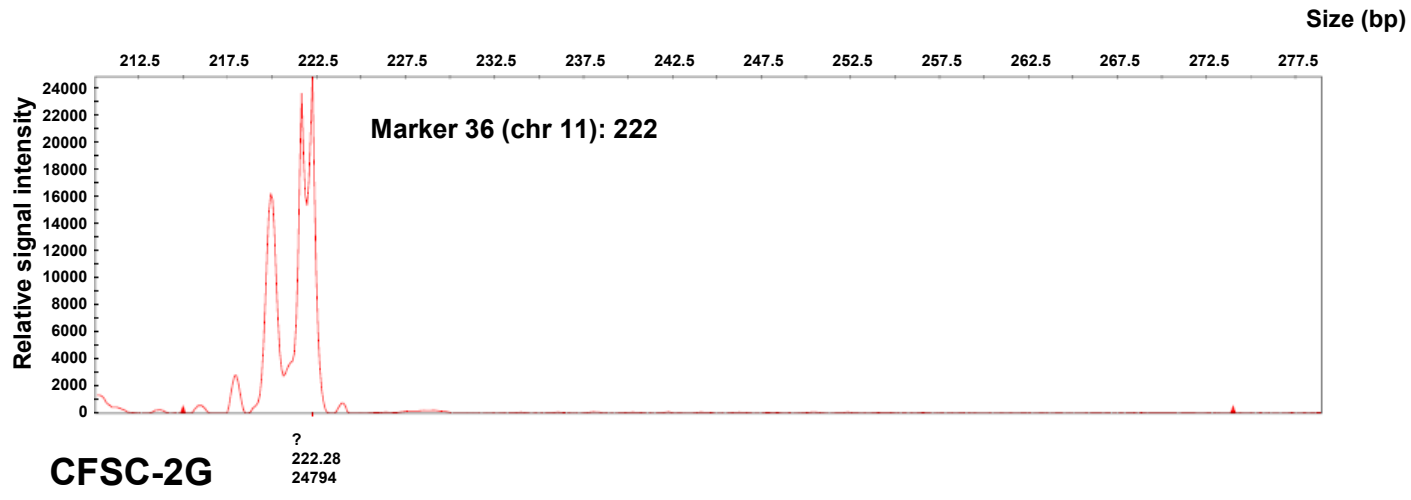

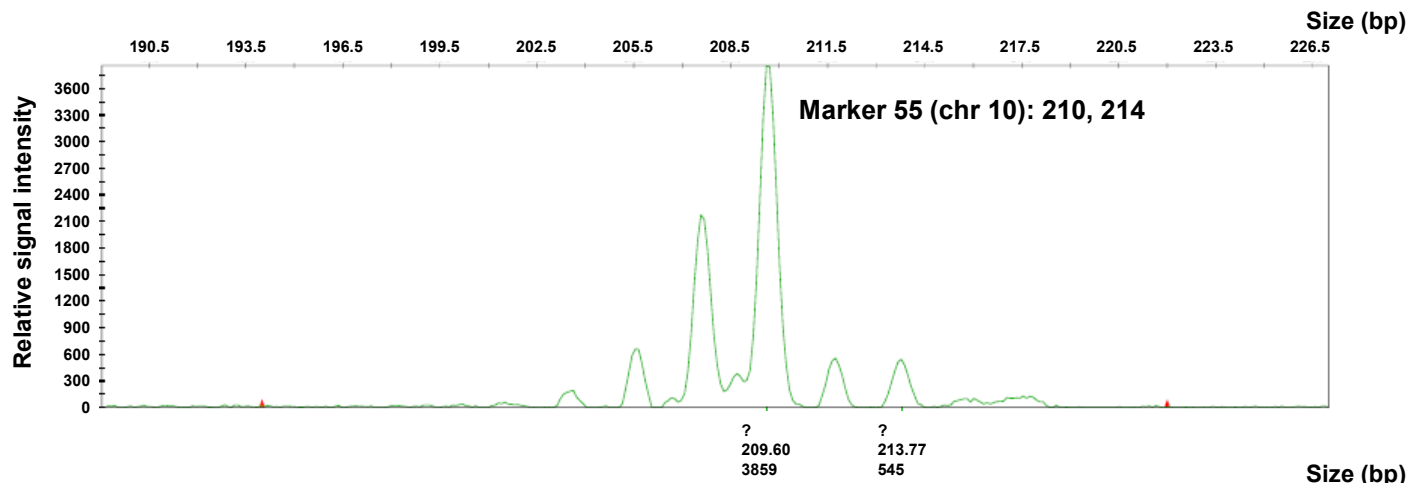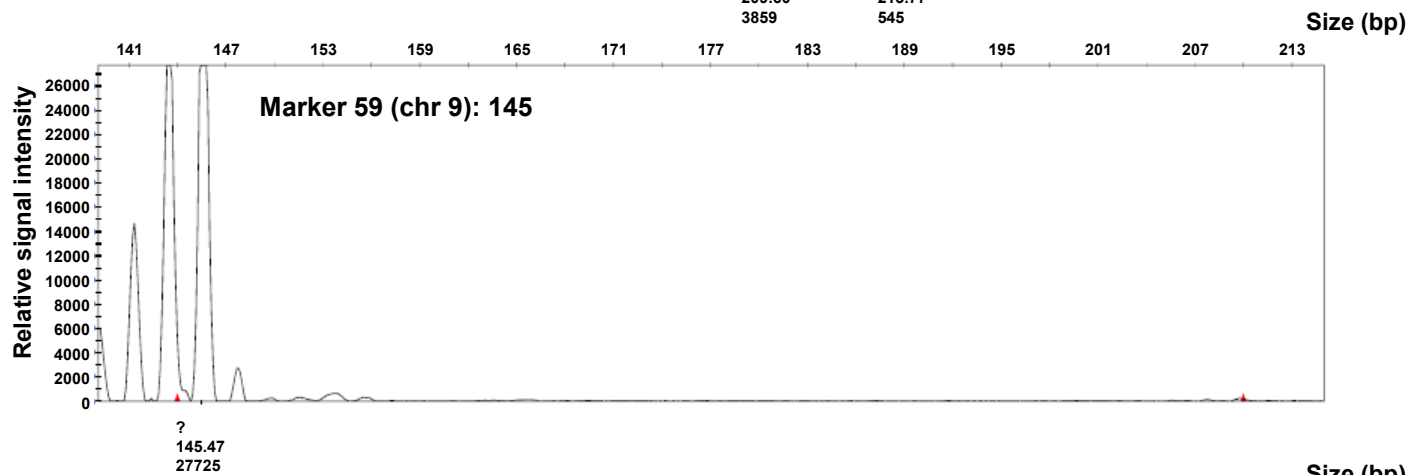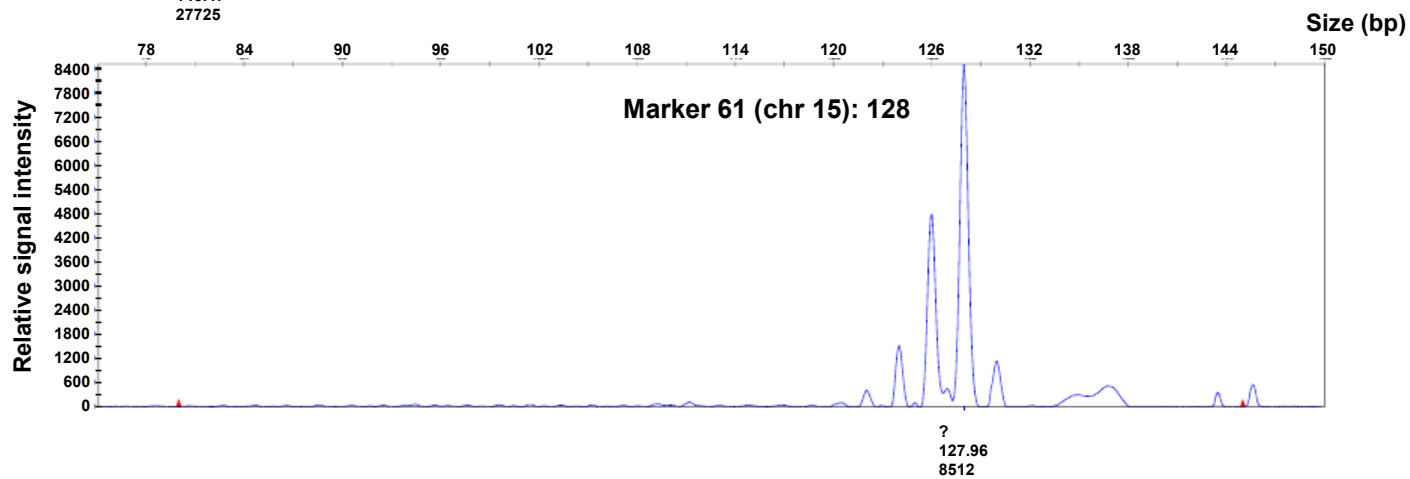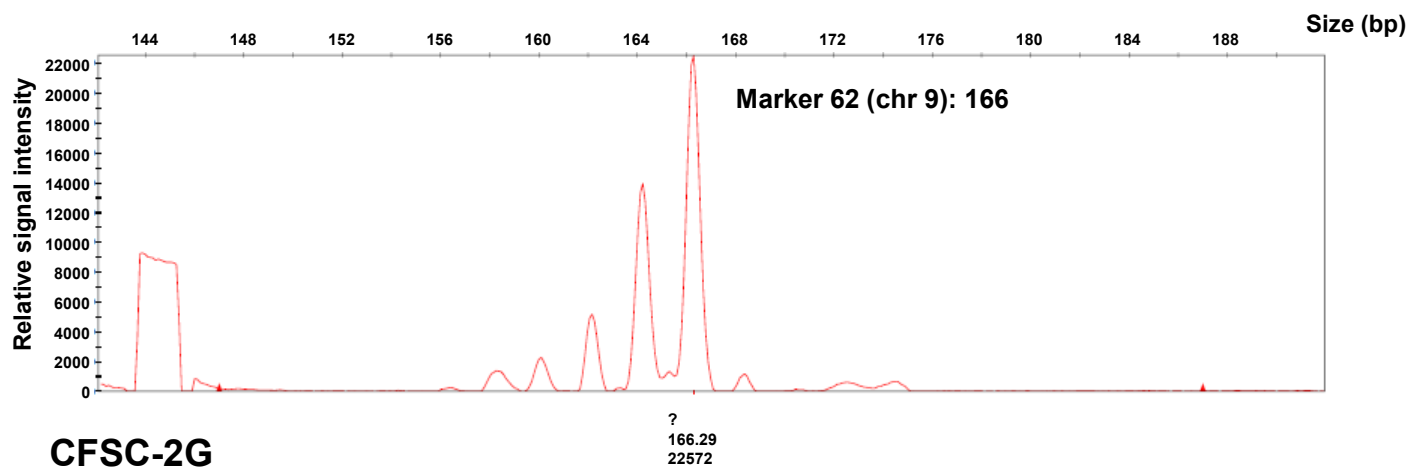

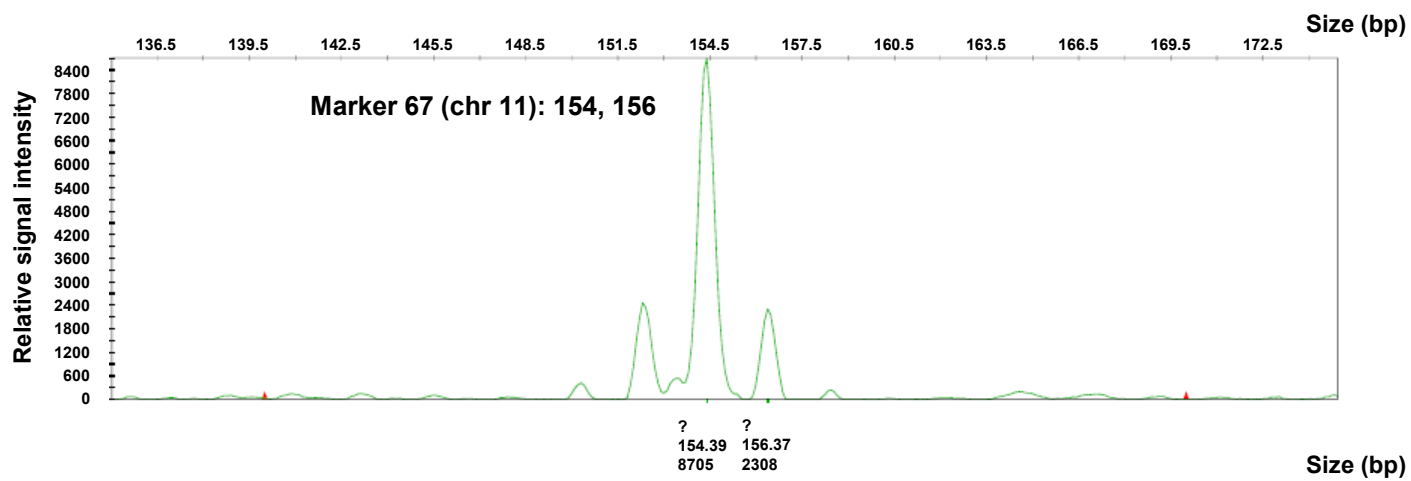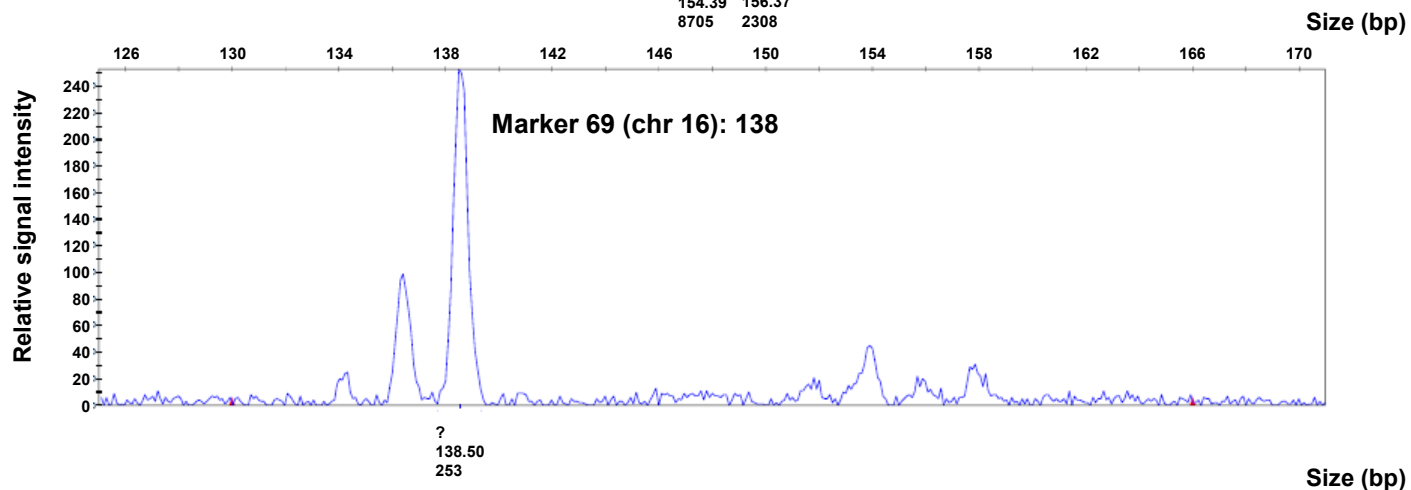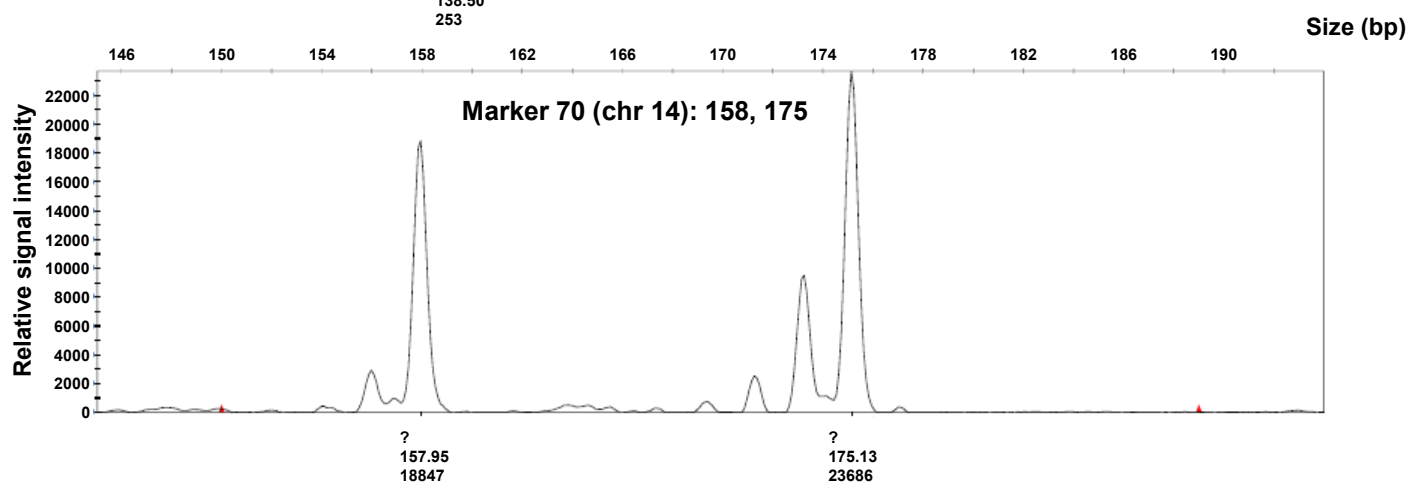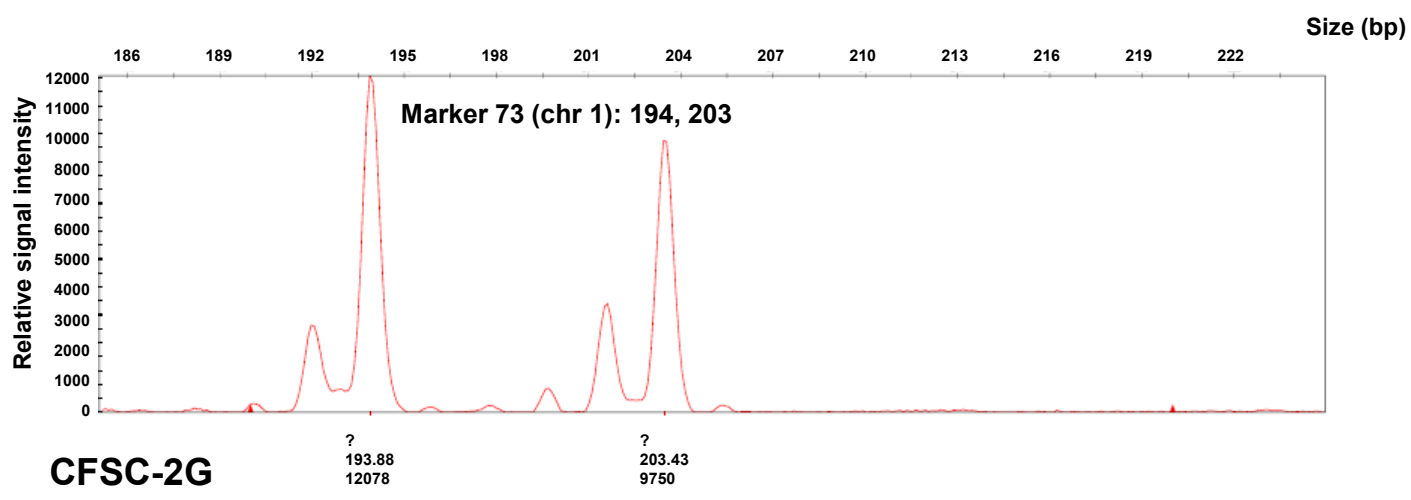

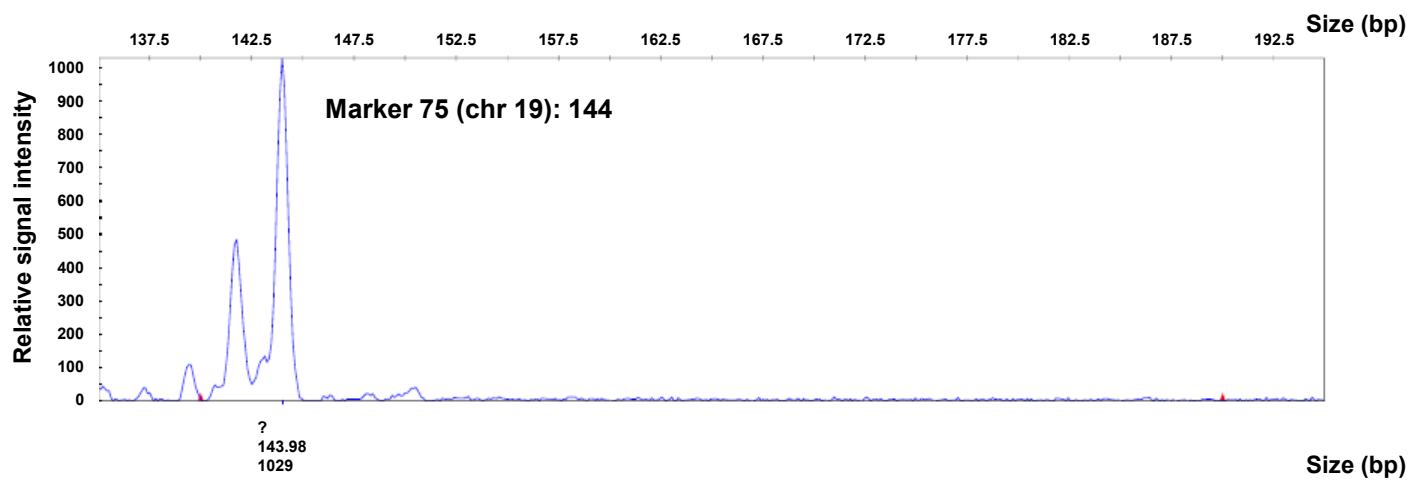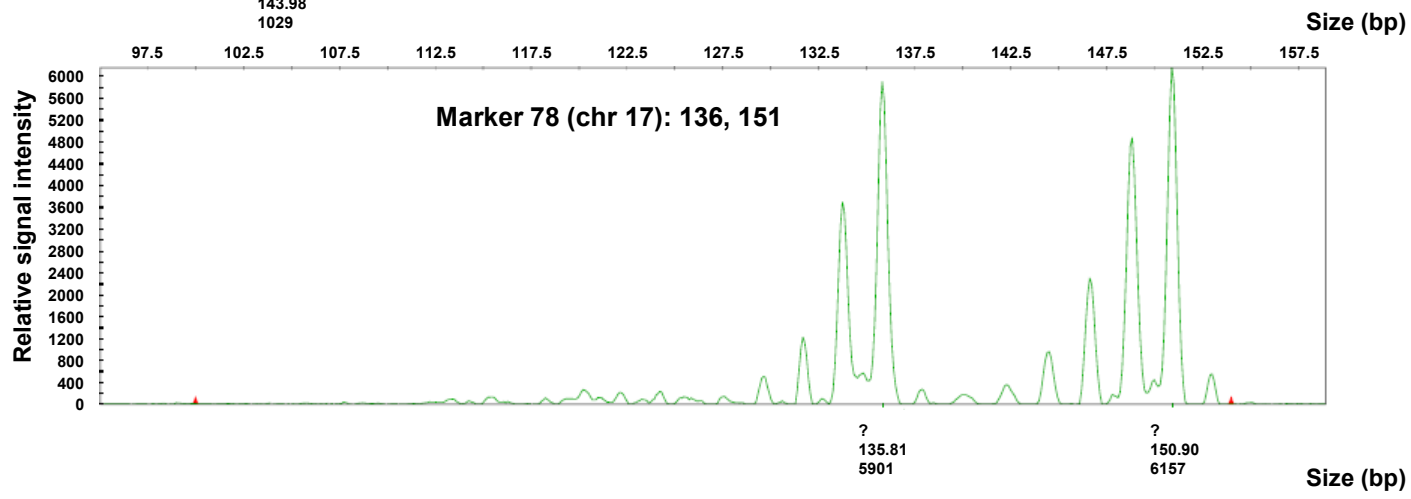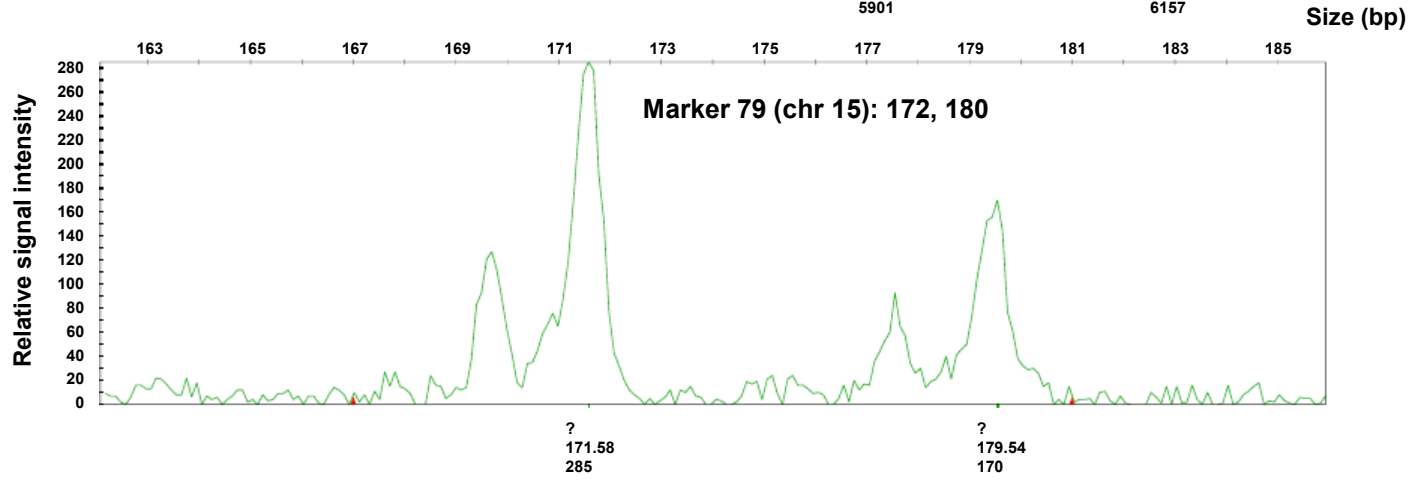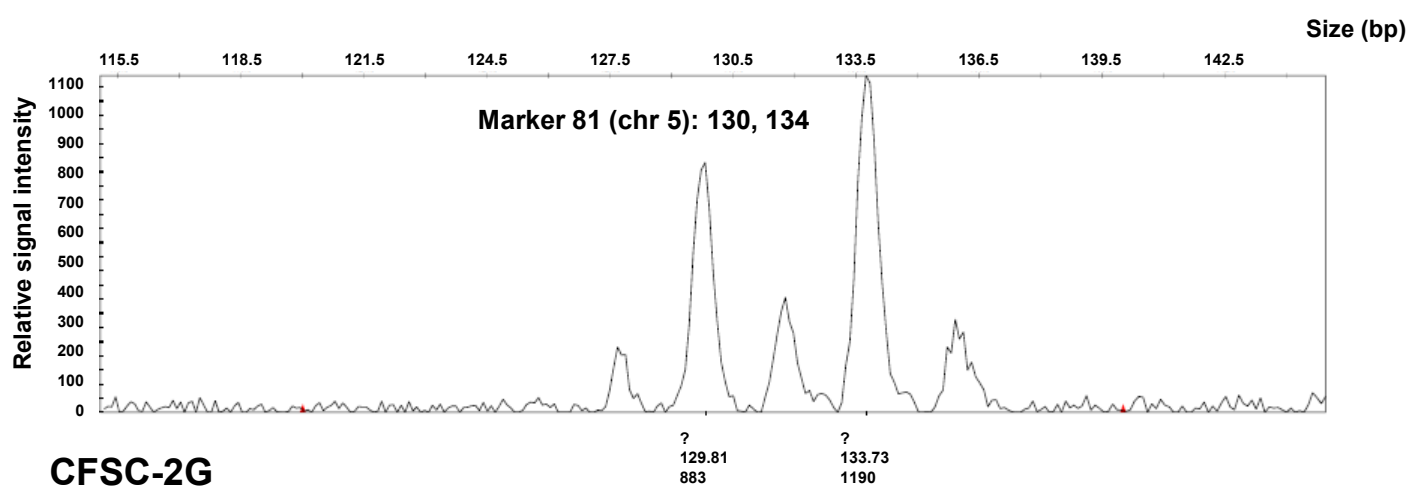

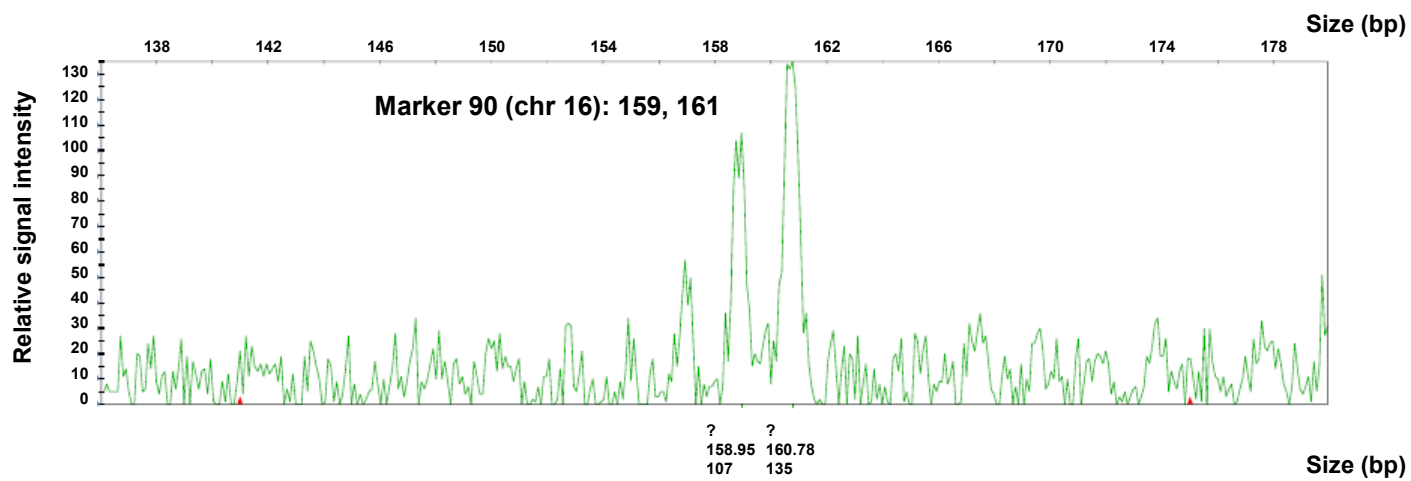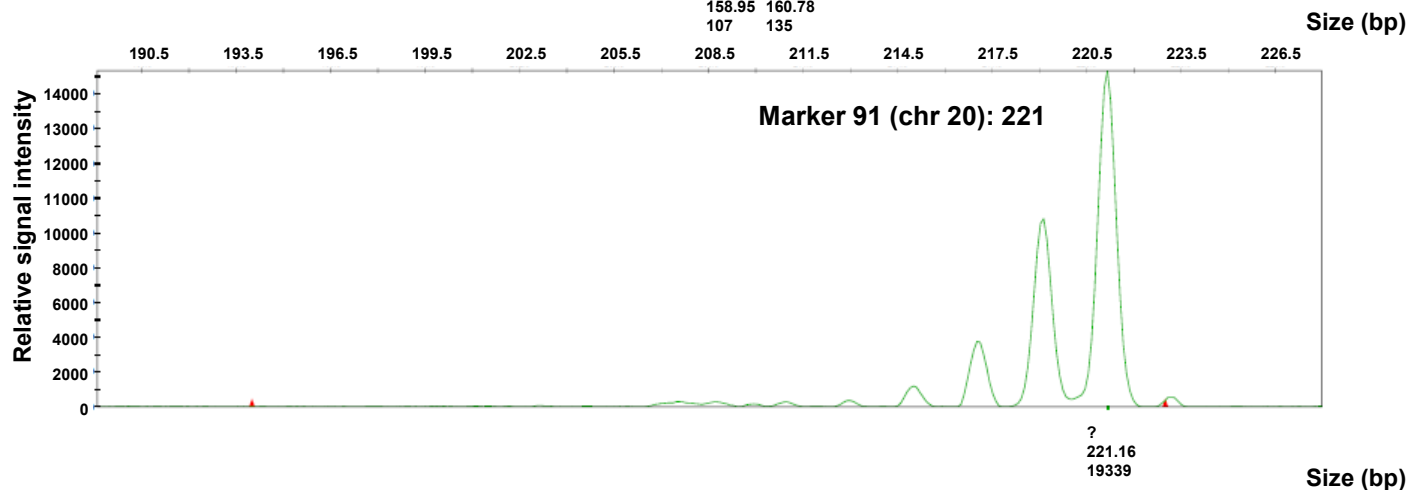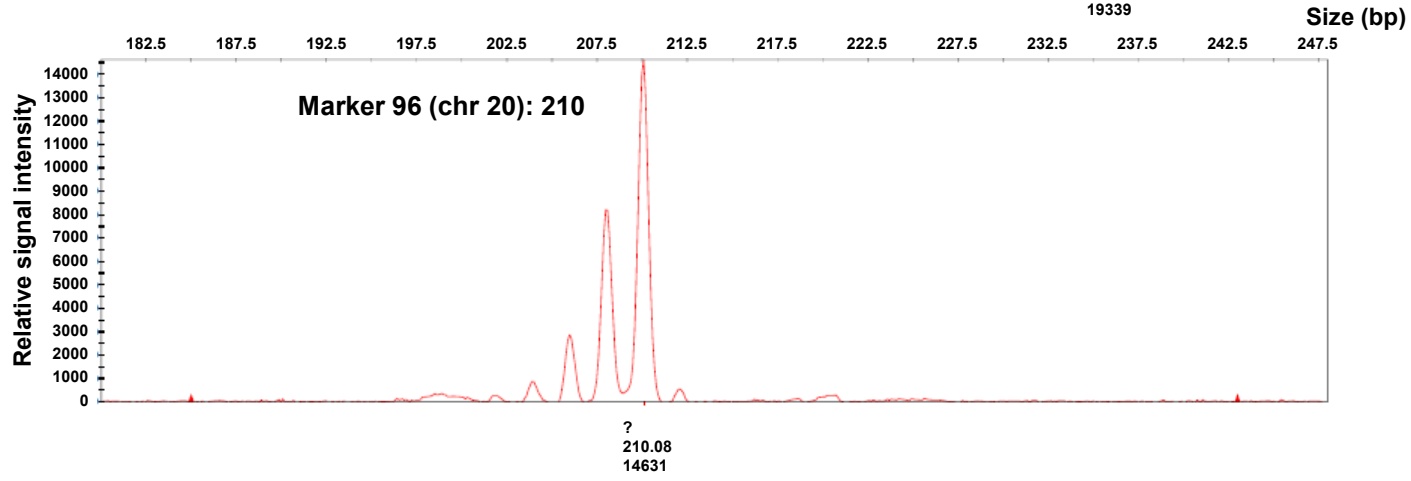

Supplement: Supplementary file 1 [file cells-11-02900-s001.zip › Figure S1.pdf]
